# Two Consecutive Cases of Anencephalus

**Published:** 1908-12-19

**Authors:** G. L. Ranking


### The General Practitioner's Column.

[Contributions to this Column are invited, and if accepted will be paid for.]

### TWO CONSECUTIVE CASES OF ANENCEPHALUS.

By G. L. Ranking, B.A.Cantab., M.R.C.S.Eng., L.R.C.P.Lond.

The occurrence of two cases consecutively of this condition, which is comparatively rare, seems to be worth reporting, seeing that they occurred within a month of each other, and also in the same locality.

*Case I.*—F. H., a primipara, ætat 28, was, according to her reckoning, eight months advanced in pregnancy. During the third week in May, she had felt what she described as "niggling pains," and on May 28 labour set in about 5 a.m. At 9 a.m. on that day by abdominal examination the fœtal head could be felt in the left iliac region; the back of the child was lying anteriorly and to the left. The fœtal heart could be distinctly heard. Per vaginam, the os was felt to be equal to the size of a shilling. Labour pains were infrequent, and by no means powerful.

Seen again at 12 noon, the os by this time was semi-dilated; labour pains were occurring every five minutes. By 3 p.m. the os was three-parts fully dilated, the membranes were bulging, and pains were becoming more powerful and more frequent. At 4 p.m. the membranes ruptured, and about half a pint of liquor amnii escaped. The presentation seemed like a face; a sharp ridge of bone occupied the centre of the circle formed by the dilated os. Laterally, and to the right one could feel the incompletely-formed pinna of the ear. The head was found to be quite small and could be easily moved. The second stage of labour progressed rapidly, and by 4.45 the face was on the perinæum. At 5 p.m. the child was born, and survived for five minutes. The placenta failed to come away within three-quarters of an hour, and at 6 p.m. was expressed. It was small, and very dark in colour, and towards the margin were three large cysts, containing blood clot.

The membranes were much torn, and portions of the placenta were missing; the patient's condition being good, chloroform was administered, and the uterus cleared out; the missing portions of the placenta were densely adherent and very hard. The uterus contracted down well, and the patient made an uninterrupted recovery.

*Case II.*—S. M., a multipara, ætat 36, was pregnant for the seventh time, previously having given birth to a dead full-term child six years ago, and in the intervening period had had five miscarriages at the third to the sixth month. Her last had taken place fifteen months previously.

The patient reckoned she was nearly a month overdue, and said that during this period her health had been very indifferent. Vomiting had been severe and prolonged, and when six months pregnant, she had to be medically treated for suppression of urine and symptoms of a uræmic nature. She volunteered the information that she was far stouter than at her previous full-time.

On examination, abdominal palpation gave no clue to the position of the child; the uterus was very tense and contracted very feebly while under manipulation. No fœtal sounds could be detected. Fœtal movements were said to have been felt on the day on which labour set in—June 21.

Per vaginam the os was semidilated, and the bag of membranes was easily felt; labour pains had set in about 8 a.m., and the patient was seen for the first time at 1 p.m. The pains were feeble and infrequent, and the diagnosis of excessive liquor amnii was made. No part of the fœtus could be felt at this time through the os.

Labour progressed slowly till 3.30 p.m., when the os was found three parts dilated; the membranes were then ruptured, and a "flood" of liquor amnii escaped; 8½ pints were caught in a receptacle, but a large quantity escaped. A vaginal examination was again made; the face was presenting and the head was freely movable in any direction. Labour pains now became more frequent and powerful, and the labour progressed rapidly. A much discoloured fœtus was born at 4.15 p.m., followed by another rush of liquor amnii. In this case the placenta was spontaneously expelled 15 minutes after the birth of the child; its size was small and signs of commencing decomposition were evident. Fifteen days after being delivered the patient was allowed to resume her housework; her general condition was fair, and her urine which, at delivery was loaded with albumen, contained only a trace.

Several points of interest are suggested by these two cases.

1. One can hardly suppose that locality can afford any reason for the occurrence of acephalous monsters; yet it must undoubtedly be rare to obtain two consecutive cases within three miles of each other in so short a space of time.

2. Barnes, in his "Obstetric Operations," states that labour in these cases is apt to be protracted; and quoting Curtis, of Melbourne, says "labour is always protracted, and there is more hæmorrhage." In neither of the cases referred to was labour unduly prolonged, and no doubt in case 2 the duration was due in some measure to the fact of the uterus being over-distended with liquor amnii. With regard to the quantity of the latter, Herman points out that it may be little or excessive; and that this is so is borne out by the cases quoted: in No. 1 the liquor was very scanty; whilst in No. 2 the amount was very considerably above the normal. With regard to hæmorrhage, in neither case was it more than is usual.

